# Comparison of Innovative and Traditional Cardiometabolic Indices in Estimating Atherosclerotic Cardiovascular Disease Risk in Adults

**DOI:** 10.3390/diagnostics11040603

**Published:** 2021-03-28

**Authors:** Ya-Chin Huang, Jiun-Chi Huang, Chia-I Lin, Hsu-Han Chien, Yu-Yin Lin, Chao-Ling Wang, Fu-Wen Liang, Chia-Yen Dai, Hung-Yi Chuang

**Affiliations:** 1 Department of Preventive Medicine, Kaohsiung Municipal Ta-Tung Hospital, Kaohsiung Medical University, Kaohsiung 801, Taiwan; jasimine0603@gmail.com (Y.-C.H.); linchiai@gmail.com (C.-I.L.); yik89045@gmail.com (H.-H.C.); 2 Department of Occupational & Environmental Medicine, Kaohsiung Medical University Hospital, Kaohsiung Medical University, Kaohsiung 807, Taiwan; minami05192000@gmail.com (Y.-Y.L.); florawang0913@gmail.com (C.-L.W.); hychuang@gmail.com (H.-Y.C.); 3 Master Program of Public Health, Department of Public Health, College of Heath Sciences, Kaohsiung Medical University, Kaohsiung 807, Taiwan; 4 Department of Internal Medicine, Kaohsiung Municipal Siaogang Hospital, Kaohsiung Medical University, Kaohsiung 812, Taiwan; karajan77@gmail.com; 5 Division of Nephrology, Department of Internal Medicine, Kaohsiung Medical University Hospital, Kaohsiung Medical University, Kaohsiung 807, Taiwan; 6 Faculty of Medicine, College of Medicine, Kaohsiung Medical University, Kaohsiung 807, Taiwan; 7 Department of Public Health, College of Health Science, Kaohsiung Medical University, Kaohsiung 807, Taiwan; 8 Hepatobiliary Division, Department of Internal Medicine, Kaohsiung Medical University Hospital, Kaohsiung Medical University, Kaohsiung 807, Taiwan

**Keywords:** Chinese visceral adiposity index, atherosclerotic cardiovascular disease, cardiometabolic index, adult

## Abstract

This study aimed to investigate the performance of innovative and traditional cardiometabolic indices, including body mass index (BMI), waist circumference (WC), Chinese visceral adiposity index (CVAI), visceral adiposity index, lipid accumulation product, a body shape index (ABSI), body roundness index, conicity index (CI), triglyceride-glucose (TyG) index, TyG-BMI, and TyG-WC, in estimating atherosclerotic cardiovascular disease (ASCVD) risk in 3143 Taiwanese adults aged 20–79 years. Elevated 10-year ASCVD risk was defined as ≥7.5% using the Pooled Cohort Equations. The performance of different indices in estimating elevated ASCVD risk was assessed by receiver operating characteristic (ROC) curves. In multivariate-adjusted logistic regression analyses, all cardiometabolic indices (*p*-value < 0.001) were significantly associated with elevated ASCVD risk in both genders, except for ABSI and CI in women. In particular, CVAI had the largest area under the curve (AUC) in men (0.721) and women (0.883) in the ROC analyses. BMI had the lowest AUC in men (0.617), while ABSI had the lowest AUC in women (0.613). The optimal cut-off value for CVAI was 83.7 in men and 70.8 in women. CVAI performed best among various cardiometabolic indices in estimating elevated ASCVD risk. CVAI may be a reliable index for identifying people at increased risk of ASCVD.

## 1. Introduction

Although therapeutic techniques and prevention strategies advance in recent decades, atherosclerotic cardiovascular disease (ASCVD) remains the major leading cause of death worldwide [1,2]. The increasing prevalence of diabetes, hypertension, dyslipidemia, overweight, and sedentary lifestyle as the global economy grows is believed to be the key elements contributed to ASCVD [3,4]. Among these factors, central obesity increases the cardiovascular risk through insulin resistance, secretion of adipokines, and pro-inflammatory proteins, leading to atherogenic endothelial dysfunction [5,6,7]. Central obesity and fat mass are modifiable factors and potentially treatment targets for reducing the burden of ASCVD [8].

Body mass index (BMI) has been widely used as a surrogate for overweight and obesity and was associated with lifetime cardiovascular disease across diverse populations [9,10]. Since BMI has its limitation of describing the distribution of the body fat [11], waist circumference (WC) can reflect visceral and central obesity better than BMI [12]. The pattern of fat distribution was recently recognized a stronger predictor than traditional anthropometric parameters for ASCVD [13]. Various novel anthropometric and cardiometabolic indices have been increasingly developed in recent years to better describe visceral obesity and body composition.

Chinese visceral adiposity index (CVAI) is a novel surrogate for assessment of metabolic health and prediction of diabetes [14,15]. Visceral adiposity index (VAI) is a marker of adipose function and distribution, and independently associated with the 10-year ASCVD incidence [16,17]. Lipid accumulation product (LAP) is associated with insulin resistance and cardiovascular disease, reflecting the central fat accumulation [18,19]. A body shape index (ABSI) and body roundness index (BRI) as well as conicity index (CI) were developed for better discriminative capacity of abdominal adipose tissue and body fat percentage, and were practical cardiometabolic indicators in previous studies [20,21,22]. Triglyceride-glucose (TyG) index, TyG-BMI, and TyG-WC are emerging indicators for insulin resistance, diabetes, and ischemic stroke [23,24,25,26]. Taken together, these traditional and novel cardiometabolic indices have their important roles linking with cardiovascular disease and risk. However, the comparison of performance of these indices in estimating elevated 10-year ASCVD risk remains uncertain. Therefore, this study aimed to investigate the ability in estimation of elevated ASCVD risk and the optimal cut-off value among the cardiometabolic indices in adults.

## 2. Materials and Methods

### 2.1. Study Design and Participants

**Figure 1:**
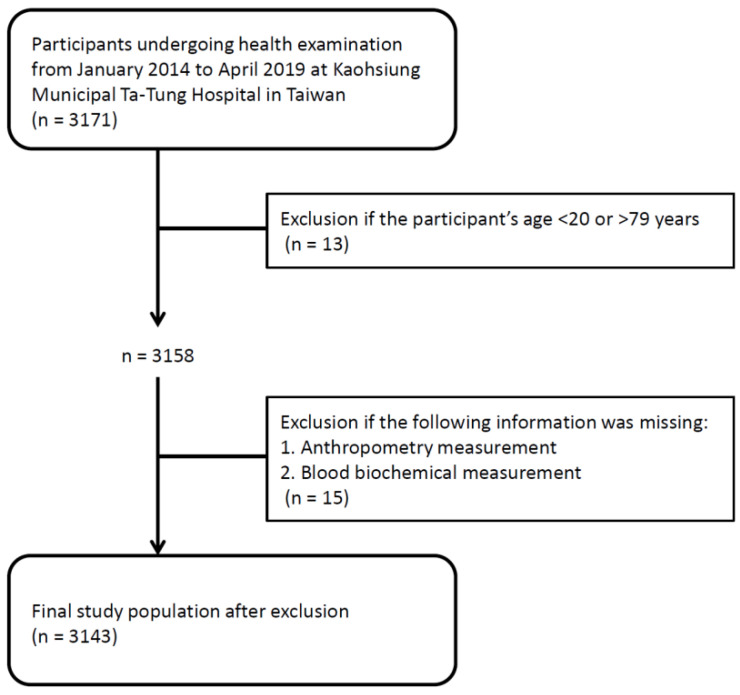
Study population and flowchart.

This study enrolled 3171 individuals undergoing health examination from January 2014 to April 2019 at a regional hospital in Taiwan. Thirteen participants aged <20 or >79 years were excluded from the study because ASCVD risk estimation is applicable for individuals with the age between 20 and 79 years [27,28]. In addition, fifteen participants with missing anthropometry or blood biochemical measurements were also excluded. Finally, a total number of 3143 adults were included in this study (Figure 1). Written informed consent has been received from each of the individuals. The study protocol was approved by the Institutional Review Board of Kaohsiung Medical University Hospital. The methods were carried out in accordance with the approved guidelines.

### 2.2. Demographic Information and Biochemical Data

Demographic information, including age, gender, smoking habits, and comorbid conditions, was obtained by the interview of study participants with the physicians. Diabetes mellitus, hypertension, and dyslipidemia were defined by self-reported history and medical history of taking anti-diabetic, anti-hypertensive, and lipid-lowering drugs from the study participants. Overnight fasting blood samples were obtained from each participant for biochemical measurements, including fasting glucose, total cholesterol, triglycerides (TG), high density lipoprotein cholesterol (HDL-C), low density lipoprotein cholesterol (LDL-C), uric acid, high sensitivity C-reactive protein (hs-CRP), and serum creatinine. Study participants’ kidney function was assessed by the estimated glomerular filtration rate (eGFR) using Chronic Kidney Disease Epidemiology Collaboration (CKD-EPI) equation [29]. Proteinuria of ≥1+ detected by a dipstick urinalysis was defined as a positive result.

### 2.3. Anthropometry Measurement, Anthropometric and Cardiometabolic Indices

Blood pressure was obtained using standard sphygmomanometers in sitting positions after taking rest at least 3 min. Body height in meter and weight in kilogram were measured according to standard methods. BMI was defined as body weight divided by the square of height. The WC was measured at the meddle point between the bottom of the rib cage and the uppermost border of the iliac crests at the end of exhalation in standing positions using an inelastic tape.

The anthropometric and cardiometabolic indices used in this study were listed as follows:

CVAI was calculated as [14]:

CVAI in men: −267.93 + 0.68 × age + 0.03 × BMI + 4.00 × WC + 22.00 × Log_10_TG − 16.32 × HDL-C

CVAI in women: −187.32 + 1.71 × age + 4.23 × BMI + 1.12 × WC + 39.76 × Log_10_TG − 11.66 × HDL-C

VAI was calculated using the following formula [16]:

VAI in men = (WC (cm)/39.68 + 1.88 × BMI (kg/m^2^)) × (TG (mmol/L)/1.03) × (1.31/HDL-C (mmol/L)); and (WC (cm)/36.58 + 1.89 × BMI (kg/m^2^)) × (TG (mmol/L)/0.81) × (1.52/HDL-C (mmol/L)) in women.

LAP was calculated as (WC (cm) − 65) × (TG (mmol/L)) in men, and (WC (cm) − 58) × (TG (mmol/L)) in women [30].

ABSI was calculated as WC/(BMI^2/3^ × height^1/2^) [31]

BRI was calculated as 364.2 − 365.5 [1 − π^−2^ WC^2^(m) Height^−2^ (m)]^1/2^ [32].

CI was calculated as 0.109^−1^ WC (m) [Weight (kg)/Height (m)]^−1/2^ [33].

TyG index was calculated as Ln (TG (mg/dL) × fasting glucose (mg/dL)/2) [34].

TyG-BMI was calculated as TyG index × BMI, while TyG-WC was calculated as TyG index × WC [35].

### 2.4. Definition of 10-Year ASCVD Risk

Each participant’s 10-year ASCVD risk was assessed using the Pool Cohort Equations, which include age, gender, race, total cholesterol, HDL-C, systolic blood pressure, diabetes mellitus, and current smoking status as variables [36]. A 10-year risk ≥7.5% was defined as an elevated risk for ASCVD, defined as nonfatal myocardial infarction or coronary heart disease death, nonfatal, or fatal stroke [37].

### 2.5. Statistical Analyses

All statistical analyses were performed using SPSS v.20.0 (SPSS Inc., Chicago, IL, USA) for Windows. Data are expressed as percentages for categorical variables, mean ± standard deviation for continuous variables with approximately normal distribution, or median (25th–75th percentile) for continuous variables with skewed distribution, such as TG and hs-CRP levels. The study participants were stratified into 10-year ASCVD risk <7.5% and ≥7.5%. We analyzed the differences between these two groups by using the chi-square test for categorical variables and the independent *t*-test for continuous variables. The univariate and multivariate-adjusted logistic regression analyses were performed to investigate the associations among the anthropometric and cardiometabolic indices with elevated 10-year ASCVD risk. Receiver operating characteristic (ROC) curves analyses were carried out to assess the performance of different anthropometric and cardiometabolic indices in estimating elevated ASCVD risk. Comparison of area under curves (AUCs) was performed using the DeLong method. The optimal cut-off values of these anthropometric and cardiometabolic indices were determined by the highest Youden index value (sensitivity + specificity − 1). A *p*-value < 0.05 was considered statistically significant.

## 3. Results

A total number of 3143 adults (1348 men and 1795 women, mean age 48.4 ± 9.2 years) were included in this study. There were 1052 (33.5%) adults had 10-year ASCVD risk ≥7.5%. The characteristics of study participants are showed in Table 1. Men with an ASCVD risk ≥7.5% were more likely to be older, have a higher prevalence of diabetes, hypertension, current smoker and proteinuria, higher blood pressure levels, BMI, WC, CVAI, VAI, LAP, BRI, CI, TyG index, TyG-BMI, and TyG-WC, higher levels of fasting glucose, total cholesterol, LDL-C, TG, hs-CRP, and lower levels of HDL-C and eGFR compared to men with an ASCVD risk <7.5%. The comparisons of characteristics between women with an ASCVD risk ≥7.5% and <7.5% were similar in men, except for the lack of significant differences in the prevalence of current smoker and proteinuria, and higher level of uric acid in women with an ASCVD risk ≥7.5%.

Table 2 displays the associations among cardiometabolic indices with elevated 10-year ASCVD risk. In the unadjusted logistic regression, all 11 cardiometabolic indices (all *p*-value < 0.001) were significantly associated with elevated 10-year ASCVD risk in both genders. In multivariate analyses adjusted for age, diabetes, hypertension, dyslipidemia, current smoking, LDL-C, uric acid, hs-CRP, eGFR and proteinuria, all 11 cardiometabolic indices (all *p*-value < 0.001) remained significantly associated with elevated 10-year ASCVD risk in men and women, except for ABSI (*p*-value = 0.924) and CI (*p*-value = 0.061) in women.

**Figure 2:**
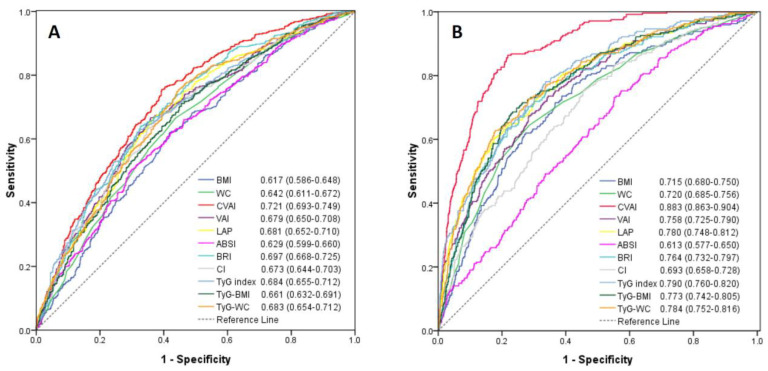
Receiver-operating characteristic curves of anthropometric and cardiometabolic indices for estimating 10-year ASCVD risk ≥7.5% in men (**A**) and women (**B**). There was significant difference of the area under curve for each anthropometric and cardiometabolic index (all *p*-value < 0.05) when compared with CVAI using the DeLong method.

Among the 11 cardiometabolic indices, CVAI had the highest AUC value in estimating an elevated 10-year ASCVD risk in men (AUC = 0.721, 95% confidence interval, 0.693–0.749) and in women (AUC = 0.883; 95% confidence interval, 0.863–0.904). The lowest AUC among these cardiometabolic indices was BMI (AUC = 0.617; 95% confidence interval, 0.586–0.648) in men and ABSI (AUC = 0.613; 95% confidence interval, 0.577–0.650) in women (Figure 2). The predictive performance of CVAI for 10-year ASCVD risk ≥7.5% was significantly better than the other 10 cardiometabolic indices (all *p*-value < 0.05). Since the menopause may affect the occurrence of ASCVD, the performance of these cardiometabolic indices were compared in women with the age ≤50 and >50 years, respectively. CVAI also had the highest AUC value in women ≤50 and >50 years of age (Figure S1).

The sensitivity, specificity, Youden index and corresponding optimal cut-off values of these cardiometabolic indices to predict an elevated ASCVD risk are demonstrated in Table 3. The optimal cut-off value of CVAI was 83.7 for men and 70.8 for women.

## 4. Discussion

Our study investigated and compared the performance of estimating elevated ASCVD risk among traditional and novel cardiometabolic indices as well as their optimal cut-off values in adults. We found that these 11 cardiometabolic indices were significantly associated with elevated ASCVD risk in both genders after adjusted for potential confounders, except for ABSI and CI in women. CVAI had the best performance in predicting elevated ASCVD risk in both men and women among traditional and novel cardiometabolic indices.

Obesity is a major challenge of public health and a common nutritional disorder worldwide. In particular, obesity has been well recognized a key factor for the development of ASCVD [38]. Emerging evidence supports that the primary culprit behind the obesity in association with cardiovascular and metabolic burden might be the pattern of fat distribution [39,40]. Although computed tomography (CT) and magnetic resonance imaging (MRI) are able to measure regional and whole body fat accurately, the lack of economic feasibility and the risk of exposure to radiation during CT scanning remain the problems for large-scale epidemiological studies. Thus, various anthropometric and cardiometabolic indices have been widely applied. In our study, the performance of estimating elevated ASCVD risk for BMI and WC were unsatisfactory compared to certain novel indices, such as CVAI, VAI, LAP, TyG index, TyG-BMI, and TyG-WC. Furthermore, gender difference was not observed with regard to the weakness for BMI and WC in prediction of 10-year ASCVD risk ≥7.5%. It indicates that BMI and WC might not be the reliable surrogate markers for identifying people with elevated ASCVD risk. Traditional anthropometric markers, BMI and WC, might marginally reflect the distribution of body fat and visceral fat mass [41].

Accumulating evidence supports that innovative indices outperform conventional anthropometric markers in relation to the incidence of ASCVD and type 2 diabetes. A high VAI is associated with elevated risk of coronary heart disease in both male and female Chinese adults [42]. VAI is independently associated with cardiovascular events among Greek adults in a 10-year follow-up study [17]. Furthermore, CVAI and VAI have better predictive ability than BMI and WC for development of diabetes in Chinese and Caucasian adults, respectively [43,44,45]. In the present study, we found that CVAI exhibited the best performance in relation to 10-year ASCVD risk ≥7.5% in both genders among anthropometric and cardiometabolic indices. Xia et al. and Wu et al. reported that CVAI is superior to VAI, BMI and WC in correlation with visceral fat area as well as the prediction of diabetes [14,15]. While visceral fat tissue has been recognized as a potential biomarker to replace the role of BMI in ASCVD risk stratification [46], excessive accumulation of visceral adiposity is supposed to have more harmful and atherogenic effects than obesity itself on the vessels [13]. Although the prospective study for investigating the link between CVAI and incidence of ASCVD has not yet published, our findings may offer a reasonable niche to advanced studies in the future.

Apart from CVAI, some novel cardiometabolic indices including LAP, TyG index, TyG-BMI, and TyG-WC, demonstrate modest performance in predicting elevated 10-year ASCVD risk. Kyrou et al. showed that LAP is a better predictor of 10-year cardiovascular events than BMI and WC [19]. Moreover, TyG index could predict the development of cardiovascular events in Caucasian and Chinese populations [47,48]. Our findings were in line with these studies, suggesting these innovative cardiometabolic indices have their pivotal roles in association with ASCVD risk. Not all the novel anthropometric markers, however, performed well in estimating elevated ASCVD risk. In Asian populations, ABSI failed to predict metabolic syndrome [49,50]. For identifying cardiovascular disease, ABSI seemed not to be a suitable predictor [20]. This might be because ABSI was generated from the US National Health and Nutrition Examination Survey 1999−2004, with three largest ethnic groups (whites, blacks, and Mexicans) included [31]. In our study, ABSI demonstrated the worst performance among the cardiometabolic indices. Taken together, ABSI may have certain weakness in relation to cardiometabolic risk in Asian adults.

The limitations of this study included an observational and cross-sectional study design. Therefore, we can only investigate the associations of various anthropometric and cardiometabolic indices with elevated ASCVD risk, but we cannot elucidate the causal relationship. Further prospective studies are needed to investigate the link between CVAI and the incidence of ASCVD. The hip circumference of each study participant was not measured in this study; therefore, the performance of estimating ASCVD risk using waist-to-hip or waist-to-height ratio cannot be compared with the other anthropometric and cardiometabolic indices. The information of the age at menopause, surgery of bilateral oophorectomy, and hormone replacement therapy was not obtained for female participants, as these factors may affect the occurrence of ASCVD. Moreover, the 10-year ASCVD risk was determined from the Pool Cohort Equations, which might not be perfectly applicable to Asian populations.

## 5. Conclusions

In summary, this study indicated that all the cardiometabolic indices were significantly associated with increased ASCVD risk in both sexes, except for ABSI and CI in women. Among the traditional and innovative cardiometabolic markers, CVAI had the best performance for estimating elevated ASCVD risk in adults. For screening and identifying people at increased risk of ASCVD, CVAI might be the relatively relevant and reliable index.

## Figures and Tables

**Table 1:** Demographic information of study participants stratified by 10-year atherosclerotic cardiovascular disease (ASCVD) risk <7.5% and ≥7.5%.

Variables	Men			Women		
	ASCVD Risk <7.5% (*n* = 538)	ASCVD Risk ≥7.5% (*n* = 810)	*p*-Value	ASCVD Risk <7.5% (*n* = 1553)	ASCVD Risk ≥7.5% (*n* = 242)	*p*-Value
Age (years)	41.7 ± 7.1	53.9 ± 7.3	<0.001	46.2 ± 8.1	58.9 ± 6.6	<0.001
Diabetes mellitus (%)	0	7.8	<0.001	0.6	14.9	<0.001
Hypertension (%)	3.0	26.4	<0.001	2.1	38.4	<0.001
Dyslipidemia (%)	1.9	2.0	0.525	1.1	0	0.151
Current smoking (%)	3.9	19.9	<0.001	0.4	0.8	0.295
Systolic BP (mmHg)	119.4 ± 11.0	129.3 ± 13.2	<0.001	115.7 ± 12.2	137.4 ± 15.0	<0.001
Diastolic BP (mmHg)	74.3 ± 9.6	80.9 ± 11.1	<0.001	70.3 ± 9.5	82.4 ± 11.0	<0.001
**Cardiometabolic Indices**						
BMI (kg/m^2^)	24.5 ± 3.3	25.9 ± 3.6	<0.001	22.4 ± 3.2	24.8 ± 3.6	<0.001
WC (cm)	84.5 ± 8.3	88.6 ± 8.8	<0.001	73.3 ± 7.4	79.5 ± 8.4	<0.001
CVAI	77.9 ± 37.6	108.2 ± 38.2	<0.001	48.0 ± 31.4	98.8 ± 29.3	<0.001
VAI	1.34 ± 1.15	2.10 ± 1.90	<0.001	1.25 ± 1.06	2.89 ± 3.60	<0.001
LAP	26.2 ± 23.5	43.2 ± 41.9	<0.001	16.8 ± 16.8	43.2 ± 47.7	<0.001
ABSI	0.076 ± 0.003	0.078 ± 0.003	<0.001	0.073 ± 0.004	0.075 ± 0.004	<0.001
BRI	3.20 ± 0.88	3.82 ± 1.01	<0.001	2.68 ± 0.85	3.60 ± 1.07	<0.001
CI	1.19 ± 0.06	1.23 ± 0.06	<0.001	1.13 ± 0.06	1.17 ± 0.07	<0.001
TyG index	9.13 ± 0.57	9.51 ± 0.60	<0.001	8.91 ± 0.53	9.58 ± 0.66	<0.001
TyG-BMI	224.6 ± 37.3	246.9 ± 42.4	<0.001	199.7 ± 35.0	238.5 ± 43.2	<0.001
TyG-WC	772.7 ± 103.3	844.3 ± 115.1	<0.001	654.2 ± 89.4	764.0 ± 113.6	<0.001
**Laboratory Data**						
Fasting glucose (mg/dL)	97.7 ± 12.3	106.6 ± 24.6	<0.001	94.8 ± 11.4	110.6 ± 27.7	<0.001
Total cholesterol (mg/dL)	196.4 ± 32.9	207.1 ± 38.4	<0.001	202.9 ± 34.3	230.2 ± 43.6	<0.001
HDL-C (mg/dL)	51.5 ± 12.2	45.4 ± 11.0	<0.001	61.6 ± 13.9	53.1 ± 13.1	<0.001
LDL-C (mg/dL)	119.7 ± 28.6	128.2 ± 33.4	<0.001	118.2 ± 30.0	139.2 ± 39.4	<0.001
Triglycerides (mg/dL)	93.0 (67.0−132.0)	130.0 (87.0−185.0)	<0.001	76.0 (55.0−107.0)	126.0 (89.0−196.5)	<0.001
Uric acid (mg/dL)	6.39 ± 1.14	6.48 ± 1.30	0.166	4.70 ± 0.92	5.38 ± 1.06	<0.001
hs-CRP (mg/L)	0.56 (0.27−1.22)	0.81 (0.40−1.79)	0.026	0.53 (0.24−1.21)	1.05 (0.55−2.56)	0.002
eGFR (mL/min/1.73 m^2^)	91.6 ± 10.4	85.3 ± 16.3	0.001	102.5 ± 20.0	97.7 ± 20.6	0.001
Proteinuria (%)	4.3	8.8	<0.001	4.0	4.5	0.726

Abbreviations: BP, blood pressure; BMI, body mass index; WC, waist circumference; CAVI, Chinese visceral adiposity index; VAI, visceral adiposity index; LAP, lipid accumulation product; ABSI, a body shape index; BRI, body roundness index; CI, conicity index; TyG, triglyceride-glucose; HDL-C, high density lipoprotein-cholesterol; LDL-C, low density lipoprotein-cholesterol; hs-CRP, high sensitivity C-reactive protein; eGFR, estimated glomerular filtration rate.

**Table 2:** Unadjusted and multivariate-adjusted odds ratios for 10-year ASCVD risk ≥7.5% among cardiometabolic indices.

Cardiometabolic Indices	Men		Women	
	Unadjusted OR (95% Confidence Interval)	Adjusted OR (95% Confidence Interval)	Unadjusted OR (95% Confidence Interval)	Adjusted OR (95% Confidence Interval)
BMI (per 1 kg/m^2^)	1.127 (1.088–1.167)	1.213 (1.129–1.303)	1.209 (1.164–1.255)	1.200 (1.115–1.291)
WC (per 1 cm)	1.060 (1.045–1.075)	1.105 (1.072–1.138)	1.096 (1.077–1.114)	1.064 (1.032–1.096)
CVAI (per 10 unit)	1.250 (1.207–1.294)	1.399 (1.297–1.510)	1.644 (1.550–1.744)	1.451 (1.316–1.601)
VAI (per 1 unit)	1.607 (1.438–1.795)	2.972 (2.380–3.713)	1.751 (1.580–1.940)	1.888 (1.612–2.212)
LAP (per 10 unit)	1.258 (1.194–1.325)	1.568 (1.414–1.739)	1.480 (1.388–1.578)	1.502 (1.358–1.661)
ABSI (per 0.01 unit)	3.959 (2.819–5.561)	3.151 (1.704–5.828)	2.533 (1.847–3.473)	1.026 (0.611–1.720) ^†^
BRI (per 1 unit)	2.178 (1.887–2.514)	2.427 (1.851–3.181)	2.458 (2.132–2.834)	1.825 (1.418–2.349)
CI (per 0.1 unit)	3.071 (2.483–3.799)	3.275 (2.200–4.874)	2.719 (2.208–3.350)	1.374 (0.985–1.916) ^‡^
TyG index (per 1 unit)	3.151 (2.554–3.889)	10.014 (6.228–16.101)	7.108 (5.408–9.343)	6.691 (4.205–10.649)
TyG-BMI (per 100 unit)	4.467 (3.252–6.135)	20.381 (9.854–42.153)	9.682 (6.837–13.710)	12.846 (6.385–25.844)
TyG WC (per 100 unit)	1.864 (1.661–2.091)	3.721 (2.812–4.923)	2.733 (2.367–3.155)	2.570 (1.967–3.359)

All *p*-value < 0.001, except for ^†^ *p*-value = 0.924, and ^‡^ *p*-value = 0.061. Multivariate adjusted for age, diabetes mellitus, hypertension, dyslipidemia, current smoking, LDL-C, uric acid, hs-CRP, eGFR, and proteinuria.

**Table 3:** Sensitivity, specificity, and Youden index using cut-off values for cardiometabolic indices to predict 10-year ASCVD risk ≥7.5%.

Cardiometabolic Indices	Men				Women			
	Cut-Off Value	Sensitivity (%)	Specificity (%)	Youden Index	Cut-Off Value	Sensitivity (%)	Specificity (%)	Youden Index
BMI (kg/m^2^)	24.6	62.0	58.2	0.202	22.7	71.5	63.3	0.348
WC (cm)	84.8	66.5	56.1	0.226	76.8	64.5	71.4	0.359
CVAI	83.7	75.6	60.6	0.362	70.8	86.3	78.2	0.645
VAI	1.23	66.7	64.1	0.308	1.26	73.6	65.6	0.392
LAP	23.9	67.0	62.8	0.298	25.5	60.3	82.5	0.428
ABSI	0.078	50.4	70.0	0.204	0.073	73.1	44.8	0.179
BRI	3.41	64.0	67.3	0.313	3.01	70.2	71.7	0.419
CI	1.20	67.9	60.6	0.285	1.13	75.2	54.6	0.298
TyG index	9.33	62.8	67.7	0.305	9.10	78.1	66.3	0.444
TyG-BMI	222.9	71.1	54.5	0.256	218.4	68.6	76.7	0.453
TyG-WC	770.2	75.6	55.4	0.310	726.3	62.8	82.0	0.448

## Data Availability

The data presented in this study are available on request from the corresponding author.

## Supplementary Materials

Supplementary materials can be found at https://www.mdpi.com/article/10.3390/diagnostics11040603/s1. Figure S1: Receiver-operating characteristic curves of anthropometric and cardiometabolic indices for estimating 10-year ASCVD risk ≥7.5% in women with the age ≤50 years (A) and the age >50 years (B).
