# Supplementary material for: Comparison of Innovative and Traditional Cardiometabolic Indices in Estimating Atherosclerotic Cardiovascular Disease Risk in Adults
Source: Diagnostics (Basel). 2021 Mar 28;11(4):603. doi: 10.3390/diagnostics11040603 (PMC8067018; doi:10.3390/diagnostics11040603)
Supplement: Supplementary file 1 [file diagnostics-11-00603-s001.pdf]

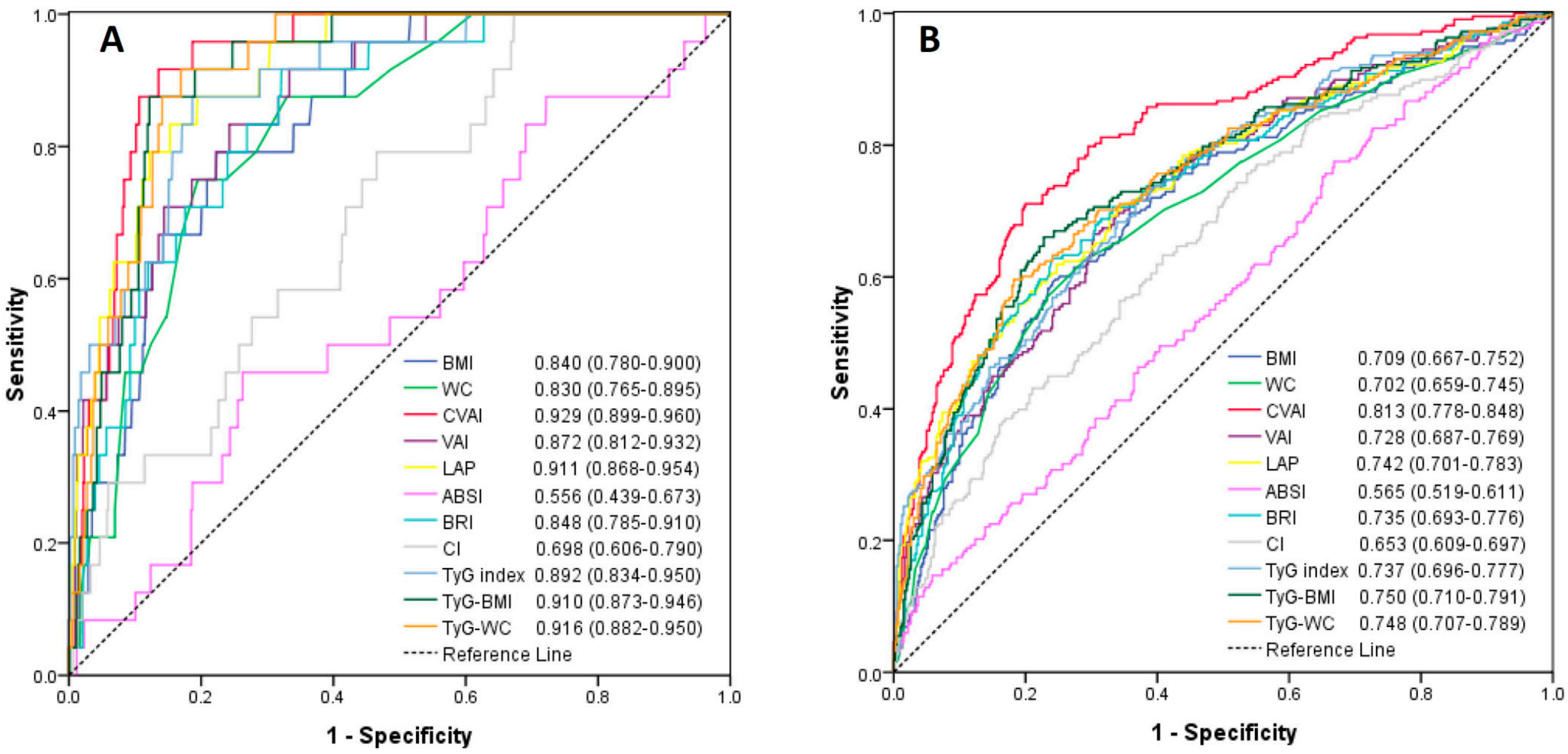

Figure S1. Receiver-operating characteristic curves of anthropometric and cardiometabolic indices for estimating 10-year ASCVD risk ≥7.5% in women with the age ≤50 years (**A**) and the age >50 years (**B**).
